# Synchronization of Kuromoto Oscillators on Simplicial Complexes: Hysteresis, Cluster Formation and Partial Synchronization

**DOI:** 10.3390/e27030233

**Published:** 2025-02-24

**Authors:** Samir Sahoo, Neelima Gupte

**Affiliations:** 1 Department of Theoretical Physics, Tata Institute of Fundamental Research, Mumbai 400088, India; samirkumsahoo@gmail.com; 2 Department of Physics, Indian Institute of Technology, Madras, Chennai 600036, India

**Keywords:** synchronization, simplices, hysteresis

## Abstract

The analysis of the synchronization of oscillator systems based on simplicial complexes presents some interesting features. The transition to synchronization can be abrupt or smooth depending on the substrate, the frequency distribution of the oscillators and the initial distribution of the phase angles. Both partial and complete synchronization can be seen as quantified by the order parameter. The addition of interactions of a higher order than the usual pairwise ones can modify these features further, especially when the interactions tend to have the opposite signs. Cluster synchronization is seen on sparse lattices and depends on the spectral dimension and whether the networks are mixed, sparse or compact. Topological effects and the geometry of shared faces are important and affect the synchronization patterns. We identify and analyze factors, such as frustration, that lead to these effects. We note that these features can be observed in realistic systems such as nanomaterials and the brain connectome.

## 1. Introduction

Complex networks can be seen in a huge variety of natural and engineered contexts. Such complex networks are characterized by quantities such as path lengths, clustering coefficients and betweenness centralities. The simplicial characterization of such networks has also revealed the existence of a hidden geometry in their structure and interactions. These networks include complex systems from the brain [1,2], designed materials [3,4,5,6] and physics problems [7,8], as well as the structures emerging from online social endeavors and large-scale social networks [9]. Such simplicial structures naturally include many-body interactions and can incorporate interactions of a higher order than the usual pairwise ones [10,11]. In the case of the dynamics of complex networks, the interplay between the dynamical evolution, the higher-order interactions and the structure of the substrate give rise to novel effects, which are a topic of recent investigations [12,13].

There are several different directions in the current research on the simplicial structure of networks. One direction is the examination of empirical data to identify the underlying simplicial structure and identify dynamical patterns via methods such as Q-analysis, persistent homology, topological information and data analysis [14,15,16,17,18,19,20]. Another direction is to investigate the dynamic and stochastic processes of these networks, to uncover the impact of higher-order structures and interactions and to identify the elements of new phenomena. To achieve this end, it is useful to model assemblies of simplicial complexes with specific structural characteristics to examine the functioning of the dynamical processes they can support. The self-assembly of growing structures using cliques is one way of constructing such structures [7,21,22]. Some striking examples of simplicial networks in the real world include (multi)-brain networks originating from human connectomes [23,24,25,26,27,28,29,30] and experimental signals [31,32]. Higher-order interactions based on simplices can appear as constitutive characteristics of complex systems in a wide variety of systems across many contexts, as mentioned above.

Recent studies have focussed on generative models of high-dimensional simplicial complexes of a controlled structure. Self-assembly models of cliques of different orders were developed in Refs. [6,22], where cliques of different sizes can be involved and the assembly is controlled by two factors: geometric compatibility and chemical affinity. The chemical affinity factor controls whether the growing structures will be dense or sparse, leading to a variety of substrate networks and important dynamical effects. The geometric compatibility factor is used to identify the common faces of the existing structure where new simplices can be attached.

## 2. The Self-Assembly of Simplices

Here, we outline a model for the self-assembly of simplices The generative models employ a bottom-up approach to grow a network with simplicial complexes with a network structure determined by controlling parameters. Here, the self-assembly of cliques is constrained by the geometric compatibility and chemical affinity factors [22]. More precisely, a simplex (clique) of some size *n* between 2 to 10 is added to an existing structure by sharing one of its faces, i.e., sub-cliques of the size $n_{q}$<n, with an already existing clique in the growing structure. Here, faces of the order $q=0,1,2,\ldots q_{max}$ correspond to a single node, link, triangle, tetrahedron, etc. The order of the largest sub-clique is $q_{max}=n-1$. At a given point in time, *t*, a new clique is attached in two steps. First, the order of the face *q* to be shared with a similar face on the existing structure is determined. Then, the remaining number of new nodes $n_{a}=q_{max}-q$ are added and all the selected nodes are joined together to form the clique.

**Figure 1:**
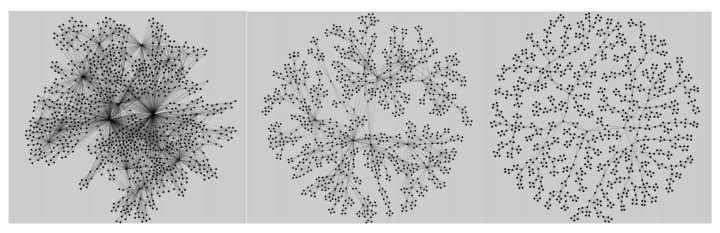
Three typical network structures obtained using the rules of self-assembly described in the text, considering the geometric compatibility and different chemical potentials ν, shown from left to right: ν= 5 (compact), ν= 0 (mixed) and ν=−5 (sparse structure) [21]. The addition of 5-cliques is stopped when the number of nodes reaches N∼ 1000.

The face that is to be shared is identified based on the geometric compatibility of a clique face with the existing structure at the given time. There is also another parameter, the chemical affinity parameter ν, which controls the number $n_{a}$ of new nodes that are added. The probability of a clique of the order $q_{max}$ attaching along the q−face is given by

(1) $$P\left(q_{max},q;t\right)=\frac{c_{q}\left(t\right)e^{-\nu (q_{max}-q)}}{\sum_{q=0}^{q_{max}-1}c_{q}\left(t\right)e^{-\nu (q_{max}-q)}}$$

where ν is the chemical affinity parameter [22] and $c_{q}$ is the number of geometrically compatible locations available for attaching a simplex of order *q* on the growing structure at time *t*. The chemical affinity factor modifies the geometric compatibility via its sign. For a large ν<0, the system favors the addition of nodes. Thus, the cliques preferably share a single node. For the opposite limit, with increasing ν>0, the cliques preferably share their larger faces, leading to compact structures. When ν=0, the process is governed by geometric compatibility alone. Consequently, each face can be shared with a finite probability, depending on its size and the actual network structure. An online demo can be seen in [33]. We note that ν=5 leads to compact structures, ν=0 leads to mixed structures and ν=−5 leads to sparse structures. The three types of networks can be seen in Figure 1. Since these have distinctly different substrates, the hysteresis effects observed for each type are different, even when other parameters are identical.

## 3. Simplicial Structure of Interactions: Higher-Order Interactions

The simplicial structures which are achieved after self-assembly provide useful and natural substrates to support higher-order interactions. Such effects have been analyzed in a variety of contexts, including spin systems and oscillator systems.

Here, we discuss continuous oscillator systems based on networks grown by the generative algorithm described above, using 5-simplex structures as the base geometry. Here, we discuss pairwise and higher interactions. We note here that the nature of the substrate, as well as the chemical affinity parameter, has its own effect on the hysteresis behavior, the transition, in this case the synchronization transition, and the patterns which appear in the system.

### 3.1. Phase Synchronization with Competing Interactions

We consider an ensemble of *N*-coupled Kuramoto oscillators associated with the nodes of a given simplicial complex [12,21]. The equation governing the evolution of the phase of *i*-th oscillator is given by [12]

(2) $${\dot{\theta }}_{i}=\omega_{i}+\frac{K_{1}}{k_{i}^{\left(1\right)}}\sum_{j=1}^{N}A_{ij}\sin\left(\theta_{j}-\theta_{i}\right)+\frac{K_{2}}{2k_{i}^{\left(2\right)}}\sum_{j=1}^{N}\sum_{l=1}^{N}B_{ijl}\sin\left(\theta_{j}+\theta_{l}-2\theta_{i}\right)$$

where ω−s represent the intrinsic frequencies of the phase oscillators. The second and third terms in Equation (2) represent 1-simplex and 2- simplex interactions, respectively. Here, $A_{ij}$ is an element of the 1-simplex adjacency matrix and $A_{ij}=1$ if nodes *i* and *j* are connected by a link, and $B_{ijl}$ is an element of the 2-simplex adjacency tensor, with $B_{ijl}=1$, if nodes i, j and *l* belong to the same triangle, and 0 otherwise. The normalization factors $k_{i}^{\left(1\right)}$ and $k_{i}^{\left(2\right)}$ indicate the number of links and triangles of the node *i*.

Thus, equal weight is given to all the terms contributing to the sum of each interaction term. In Equation (2), $\omega_{i}$ is the intrinsic frequency of the *i*-th oscillator, which governs its dynamics when there is no interaction with other oscillators in the network. The pairwise interactions seek to reduce the difference between the phase of the *i*-th oscillator and each of its neighbouring oscillators when $K_{1}>0$. In contrast, the oscillators tend towards opposite phases when $K_{1}<0$. The third term, representing three-node interactions of the *i*-th oscillator based on each 2-simplex incident on node *i*, is a natural generalization of the pairwise interaction term. It is important to note that the interactions between these three nodes occur over the faces of the simplicial complex and not over any given three nodes. Furthermore, this interaction term is symmetric in *i* in that it is unaffected by permutations in the other two indices.

Here, through synchronization, by which we mean perfect synchronization, all the phase angles at all the considered nodes evolve with identical values. We can also use cluster synchronization, where some of the nodes that are connected to each other remain in step with each other, whereas other nodes are not in step. There can be one or more synchronized clusters in the network.

The degree of synchronization of the whole network is quantified by the usual Kuramoto-order parameter, defined by

(3) $$r=\langle \left|\frac{1}{N}\sum_{j=1}^{N}e^{\iota \theta_{j}}\right|\rangle$$

Here, the angular brackets indicate the time average. Hence, r=1 represents the case of perfect synchronization, i.e., all phases are equal, and r=0 corresponds to the disordered phase. Meanwhile, the stable states with 0<r<1 indicate the presence of more complex synchronization patterns, which arise due to the competition between interactions and the effect of shared faces.

### 3.2. Effects of Interplay

In this context, we examine the emergence and disappearance of collective dynamic behavior, as measured by the order parameter on the hysteresis loop when the relative strengths of the various interactions embedded in the geometry vary. For example, the presence of triangle-based interactions is understood to disrupt the promoted order by increasing the strength of pairwise couplings and can cause abrupt desynchronization [7,34,35]. Furthermore, the occurrence of partially synchronized phases with negative pair interactions is another striking feature of geometric interactions on simplicial complexes; see, for example, [12] and references therein.

The influence of topology on diffusive processes has been examined, e.g., via a spectral analysis of the network [36,37,38,39,40,41,42,43]. One possible measure is the spectral dimension as derived from the eigenvalue spectrum of the Laplace operator associated with the network adjacency matrix. In the context of phase synchronization, it has been observed that networks with $d_{s}>4$ are required to enable stable global synchronization, while such states cannot be reached when $d_{s}<2$. There has been some analysis of the conditions for global synchronization [8,44]. However, the origin of partial synchronization on simplicial complexes and the nature of the underlying dynamical states needs further analysis. In addition to the topological dimension of a simplicial complex, the role of its architecture in the synchronization processes remains an open problem, especially in relation to by the presence of higher-order interactions and the internal inhomogeneity of the nodes’ dynamics.

An important physical consequence of the manner in which the extent to which these factors influence the transition to synchronization and desynchronization can be inferred is the behavior of the hysteresis loop. As seen above, $K_{1}$ and $K_{2}$ denote the strength of the pair and triangle interactions. The hysteresis is studied via the behavior of the order parameter, which is a measure of the level of synchronization in the system of increasing strengths in the pairwise coupling (forward sweep), as well as decreasing ones (reverse sweep) for fixed values of the triplet coupling. We examined both positive and negative values of $K_{1}$.

We examined the response of the system via the hysteresis loop of the system for different simplicial geometries, and for varying contributions from pairwise and triangular interactions. These factors have significant effects. The structure of the networks grown here is controlled via the growing algorithm, which incorporates the geometric compatibility and the chemical affinity, which essentially control the number of neighbors of each node, leading to varying effects. These include partial or frustrated synchronization and the presence of fluctuations and meta-stable states. While many aspects of the observed patterns are under analysis, we discuss the current status in the next section.

### 3.3. Hysteresis Effects

We start with the simplest case: the synchronization process using only the pairwise interactions. These interactions are enabled by the edges of the simplicial complex underlying the network via the connectivity matrix. Hence, different network features from the local to global levels are expected to play a role in the cooperative behaviors, depending on the interaction strength $K_{1}$.

For the simulations here, the initial condition for $\theta_{i}$ was chosen from the uniform distribution [0,2π] and the frequencies $\omega_{i}$ at each node *i* were chosen from a the Gaussian distribution with a zero mean, respectively. The numerical solution of the set of equations was obtained using the Python library SciPy (version 1.0 (2020)), with 50,000 iteration steps, always considering the previous state of each dynamical variable (parallel updates).

**Figure 2:**
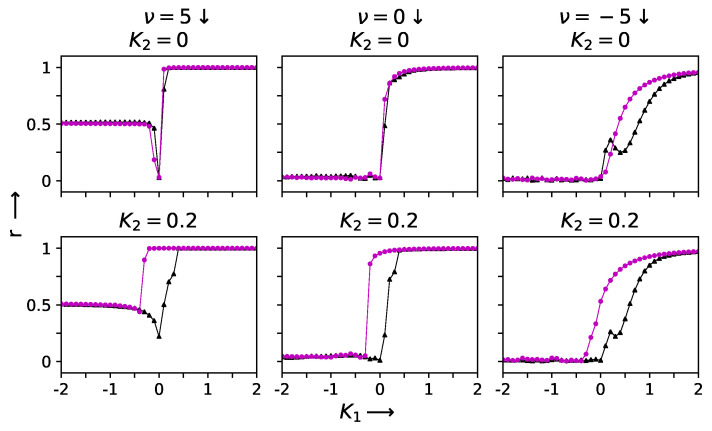
The transition to synchronization and hysteresis behavior shown by networks with ν=5, 0, −5 when ω is drawn from a Gaussian distribution with mean frequencies Ω=1 and σ= 0.01. The parameter $K_{2}$ is varied along the three columns, namely, $K_{2}=$ 0 & 0.2 for the top and bottom horizontal row panels, respectively. The solid triangles (black) and solid circles (magenta) represent the value of order parameter *r* in the forward and backward sweeps, respectively [21].

For the four-dimensional simplicial complex, as $K_{1}$ increased from zero to $K_{1}=2.0$, we observed a continuous transition from a desynchronized state where (r=0) to a completely synchronized state r=1; see Figure 2. On the other hand, as $K_{1}$ decreased from zero to $K_{2}=-2.0$, a partially synchronized state (r=0.6) was reached. The same behavior was seen for both increasing and decreasing increments in $K_{1}$. Thus, there was no hysteresis. Randomized versions of the network show a fully desynchronized regime for $K_{1}<0$.

**Figure 3:**
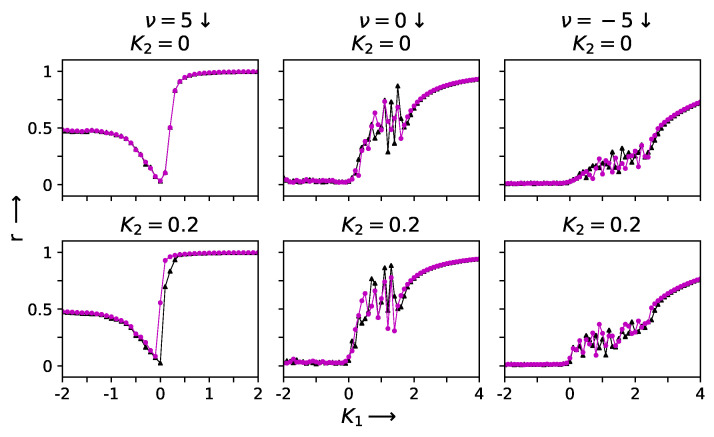
The hysteresis sweep of the order parameter *r* is plotted against $K_{1}$ for the three different networks with ν=5, 0 and −5 (along three vertical columns), at $K_{2}=$ 0 and 0.2 (along two horizontal panels). The solid triangles (black) and solid circles (magenta) represent the value of the order parameter *r* for the forward and backward sweeps, respectively. The frequency ω is drawn from a Gaussian distribution with a mean of 1 and standard deviation of 0.1 [21].

The introduction of two-simplex interactions changes the situation drastically. A hysteresis loop now opens up due to the presence of triangle-based interactions. The hysteresis loop grows in size as $K_{2}$ is increased, affecting both the forward sweep on the positive $K_{1}$ side and the size of the first-order jump. Similar behavior is seen for a wider range of values of the 2-simplex interaction strength as long as the large positive pairwise interactions are sufficient to maintain complete synchrony. However, for larger values of $K_{2}$, the fully synchronized state is no longer accessible; instead, partial synchronization is reached in the presence of the competing interactions. The detailed behavior that is observed depends on the sign and strength of the pairwise interactions. These features can be seen in Figure 2 and Figure 3.

The partial synchronization seen in the system can be attributed to frustration effects [43]. It is necessary to analyze small lattices to identify the exact frustration effects and patterns. However, cluster synchronization can clearly be discerned via the distribution of phases for these large lattices. We note that such effects have been observed in other systems and analyzed via eigenvalue analysis [45].

**Figure 4:**
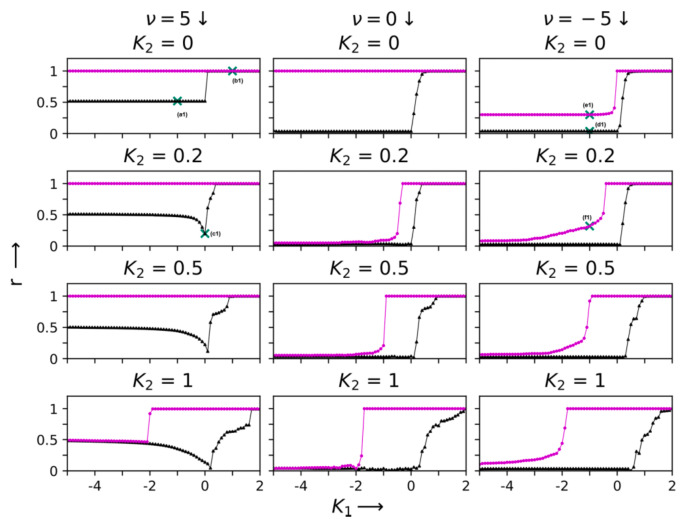
The hysteresis sweep of the order parameter *r* as a function of the one-simplex coupling strength $K_{1}$ at different two-simplex coupling strengths $K_{2}$; the three vertical columns (from **left** to **right**) correspond to the three simplicial complexes seen in Figure 1, grown with the chemical affinities ν=5, 0 & −5, respectively. In each panel, the solid triangles (black) and solid circles (magenta) refer to forward and backward sweeps, respectively. The intrinsic frequencies are $\omega_{i}=\omega =$1 at all nodes. The phase evolution patterns analyzed in the text correspond to the points indicated by crosses here [21].

It should also be noted that if all oscillators rotate with a single common frequency $\omega_{i}=\omega$, then discontinuous transitions and hysteresis behavior can be seen, even for the pure pairwise interaction case (where $K_{2}=0$) [21]. Thus, higher-order triangle-based interactions are not necessary to see a first-order transition for this case (see Figure 4).

## 4. Patterns and Dynamics of Individual Nodes

**Figure 5:**
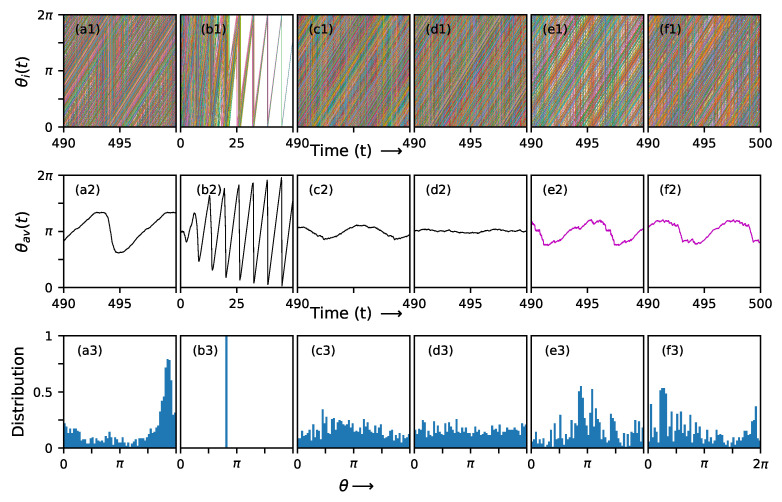
The phases of elementary nodes $\theta_{i}$ as a function of time, the average phase θ as a function of time and the distribution of phases (in radians) in the final (after 50,000 iterations) states of the simulations are shown along the three vertical panels i.e., from top to bottom, respectively, for different combinations of $K_{1}$ & $K_{2}$ when ω=1 for each node of a network. The vertical panels (**a1**–**c1**) and (**d1**–**f1**) correspond to the networks with ν=5 and −5, respectively. The ($K_{1}$, $K_{2}$) combinations along these vertical panels are the following: (**a1**–**a3**) (−1, 0)-forward (**b1**–**b3**) (1, 0)-forward, (**c1**–**c3**) (0, 0.2)-forward, (**d1**–**d3**) (−1, 0)-forward, (**e1**–**e3**) (−1, 0)-backward, (**f1**–**f3**) (−1, 0.2)-backward [21].

The hysteretic behavior discussed above was explored via the global-order parameter *r*. It is also instructive to examine the phase angle behavior at individual nodes, as well as the time evolution of the average angle, $\theta_{av}\left(t\right)=\sum_{i}\frac{\theta_{i}\left(t\right)}{N}$, and the distribution of phase angles at a fixed time *t*. These can be seen for specific values of $K_{1},K_{2}$ in Figure 5, which were selected from the forward and backward branch of the hysteresis loops for the ν values ν=+5, ν=0.0 and ν=−5.

1. ν=5, compact networks: Here, we choose the values $K_{1}=-1,K_{2}=0$ on the forward branch with a pure pairwise interaction, *f* and $K_{1}=1,K_{2}=0$, on the backward branch. The behavior of the three quantities of interest, $\theta_{i}\left(t\right)$, $\theta_{av}\left(t\right)$ and the distribution of $\theta_{i}$, can be seen in panels a1,a2,a3 of Figure 5. It can be seen from panels a1 and a3 that the $\theta_{i}$ has one prominent peak value in the distribution, with a range of $\theta_{i}$ values in the rest of the distribution. The average angle in the time evolution from 490 to 500 steps shows one clear oscillation and then flattens off.
  At $K_{1}=1$, $K_{2}=0$, we examine the first 500 steps and can hence see the transient before the iterates settle. Panel a2 shows that the $\theta_{i}$ tends to synchronize. The average angle b2 shows small transient values due to the spread of phase angles and then shows a clear oscillation. The distribution of phase angles peaks around a single value.
  At $K_{1}=0$, $K_{2}=0.2$, the $\theta_{i}$-s shows a wide spread (see panels c1 and c3). The average angle shows a reasonably regular oscillation between time steps 490 and 1000 (panel c2).
2. ν=−5, sparse networks: The behavior of the sparse network is shown in the (d,e,f) panels. The *d* and *e* panels show the behavior of the pairwise interactions with $K_{1}=-1$ on the forward and backward branch alone for a negative value of *K*. The *f* panels show the effects of the higher-order term alone, i.e., $K_{1}=0$, $K_{2}=0.2$, forward branch. The pairwise backward branch shows a banded structure in the e1 plot, which translates into oscillations in the $\theta_{av}$, as seen in e2. The forward branch shows a more or less constant value of the $\theta_{av}$. The higher-order interactions also show clear oscillations in panel f2. Hence, peaks are seen in the distribution in e3 and f3. However the higher-order interactions show a more pronounced peak.
  We note that the clustering is much more clear in the compact structure. The sparse structure tends to distribute the phase angles more uniformly across the available range, although the higher-order interactions promote some clustering. Thus, both the kind of interaction and the substrate influence the synchronization and clustering seen in this system.

The networks tend to not synchronize if only a single node is shared between the simplices and show clustered behavior as higher *q* structures, such as links, triangles and tetrahedra are shared between the structures. At K=1, the structures show full synchronization, as seen from the hysteresis loops. Switching on the $K_{2}$ interactions shows that $K_{2}=0.5$, $K_{1}=-1.0,0.4$ and 0.5 have similar behavior, with one node shared between the simplices. Sharing a triangle leads to two clusters for $K_{2}=0.5$ and both $K_{1}$ = −1.0 and 0.0. For $K_{2}=0.5$ and $K_{1}=0.0,0.3$ and 0.4, with a common link, show clustered behavior again, with the number of nodes that are synchronized increasing with an increase in the strength of pairwise interactions. Common tetrahedra with $K_{2}=0.5$ and $K_{1}=0.0$ and $K_{1}=0.3$ show two clustered behaviors.

### Partial Synchrony and Patterns

The partial synchrony seen here is a consequence of cluster formation, as can be confirmed through the behavior of larger lattices. The number of clusters that is formed decreases with the increase in the number of nodes in the common faces, as might be expected. The more compact structures have a greater tendency to synchronize globally, in contrast with the sparse structures. Negative pairwise interactions tend to inhibit or ‘frustrate’ the synchronization by pushing the phase angles apart. We will now discuss this in further detail.

We note that some studies of Kuramoto oscillators on regular two-dimensional lattices showed frustration effects [46] for the case of repulsive pairwise coupling. Here, at higher coupling parameter values, the oscillators relaxed into a state that was $\frac{2\pi }{3}$ out of phase with its neighbor. Such states are called splay states in the literature, and were observed in the context of defects [46] in oscillator systems, as well as in coupled Sine circle map lattices [47]. We note that such states lead to partial synchronization in the system.

## 5. Small Systems: Two Simplex Systems

**Figure 6:**
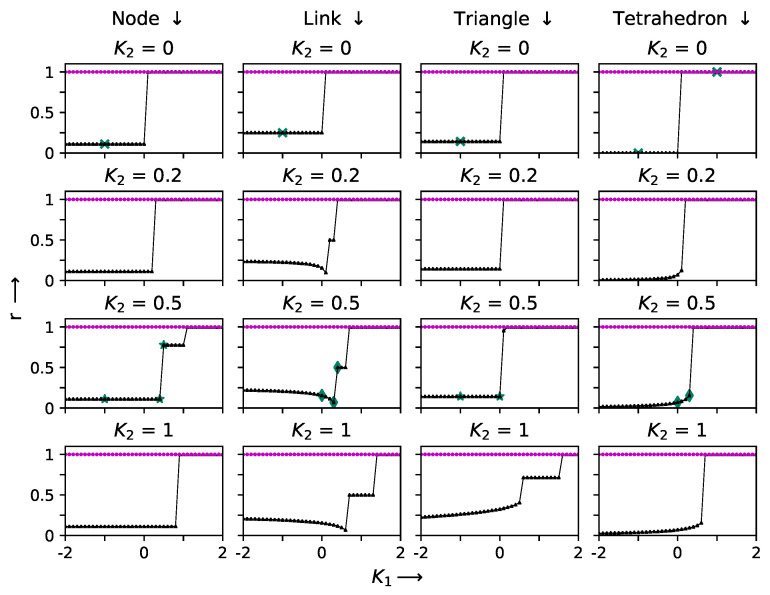
The hysteresis sweep of order parameter *r* as a function of $K_{1}$ at different values of $K_{2}$, varying along the columns; the four vertical columns (from **left** to **right**) correspond to four simplices sharing a node, link, triangle and a tetrahedron, respectively. In each panel, the solid triangles (black) and solid circles (magenta) refer to forward and backward sweeps, respectively. The intrinsic frequencies $\omega_{i}=$1 at all nodes. The evolution of phases with time is analyzed at the the points indicated by crosses, stars, and diamonds.

Small simplex systems constitute the simplest case in which the patterns can be analyzed. Here, we analyze the cases of two 5- simplex cliques for the cases where the two simplices share a node, a link, a triangle and a tetrahedron. The hysteretic behavior seen here is shown in Figure 6. The intrinsic frequency was ω=1.0 at all nodes. We noted hysteretic behavior in all cases and also stable behavior once full synchronization was reached, despite the decreasing coupling, regardless of whether pairwise or triplet interactions occurred. A partially synchronized case was observed, even in the single simplex case, for $K_{2}=0.5$. For the two simplex cases, the nine node cases (one node shared) showed partial synchronization everywhere. Desynchronization was only observed at two points in the hysteretic loops for $K_{2}=0.5,1$. The triangle shared case (eight nodes) showed partial synchronization in all cases, with a stable step for $K_{2}=0.5$. The case with shared tetrahedra showed a gentler increase to full synchronization.

**Figure 7:**
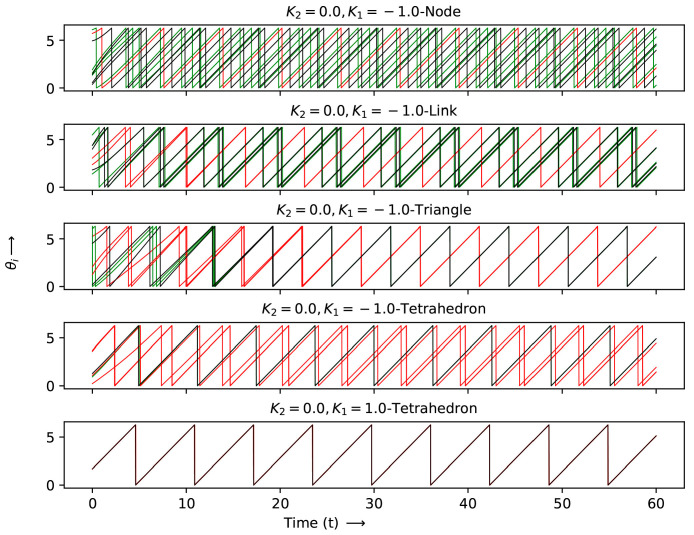
The to a distinct node. phases of each node $\theta_{i}\left(t\right)$ vs. *t* at different $K_{1}$ and $K_{2}$ values, marked by crosses in Figure 6.

**Figure 8:**
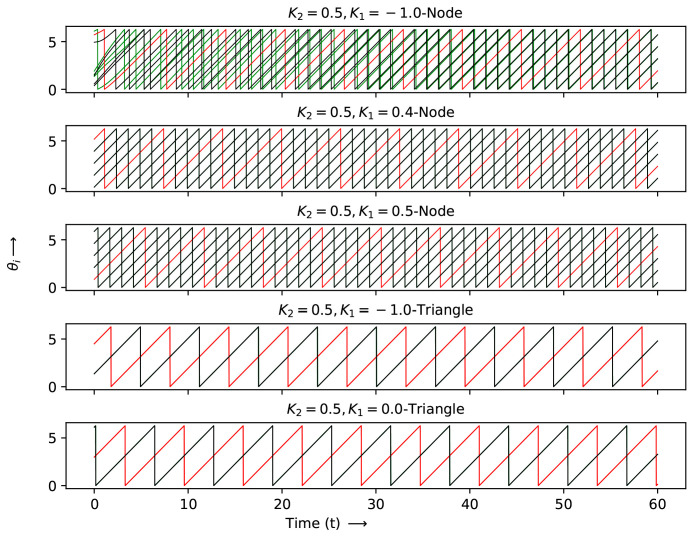
The phases of each node $\theta_{i}\left(t\right)$ vs. *t* at different $K_{1}$ and $K_{2}$ values, marked as stars in Figure 6. Each color corresponds to a distinct node.

**Figure 9:**
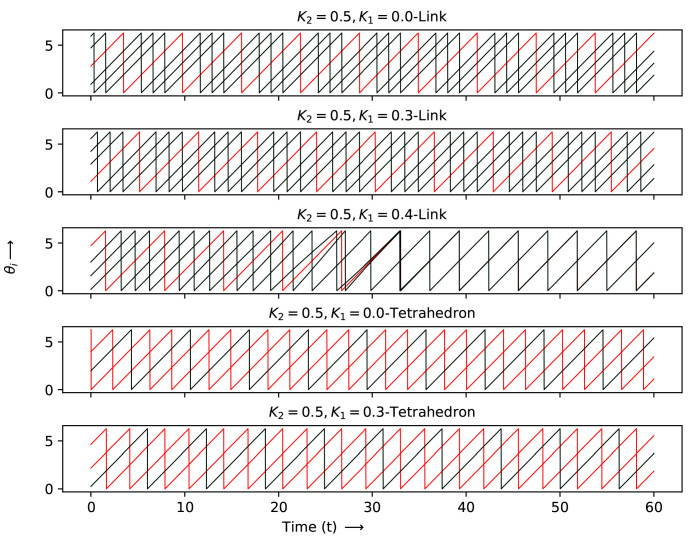
The phases of each node $\theta_{i}\left(t\right)$ vs. *t* at different $K_{1}$ and $K_{2}$ values, marked as diamonds in Figure 6. Each color corresponds to a distinct node.

The detailed evolution at each node for the two simplex case is shown in Figure 7, Figure 8 and Figure 9 for the $K_{1}$ and $K_{2}$ values indicated by crosses ($K_{2}=0.0,K_{1}=-1.0$), stars and diamonds in Figure 6:

1. Crosses ($K_{2}=0.0,K_{1}=-1.0$): Here the node with two simplices shows maximum desynchronization. The link that shared two simplices shows four clusters; the triangle that shared two simplices reduced to two clustes; the tetrahedron case showed four clusters again. The ($K_{2}=0.0,K_{1}=1.0$) case shows full synchronization.
2. The cases denoted by stars ($K_{2}=0.5;K_{1}=-1,0.4$ and 0.5) show several clusters for the node shared case and two clusters can be observed for the shared $K_{1}=-1.0,K_{1}=0$ cases denoted by a triangle.
3. Diamonds: ($K_{2}=0.5;K_{1}=0,0.3$, and 0.4 case, link shared). The first two cases are multiclustered, while the last case show two clustered solutions. The shared tetrahedra cases marked by diamonds ($K_{2}=0.5,K_{1}=0.0$ and 0.3 cases show three oscillating cluster solutions.

We note that the backward sweep is very stable, and once synchronization is achieved, the two clusters are very stable. It may be interesting to see what happens if the frequencies are no longer uniform for all nodes and are locked to specific values.

### Patterns of the Two Simplex Structure

**Figure 10:**
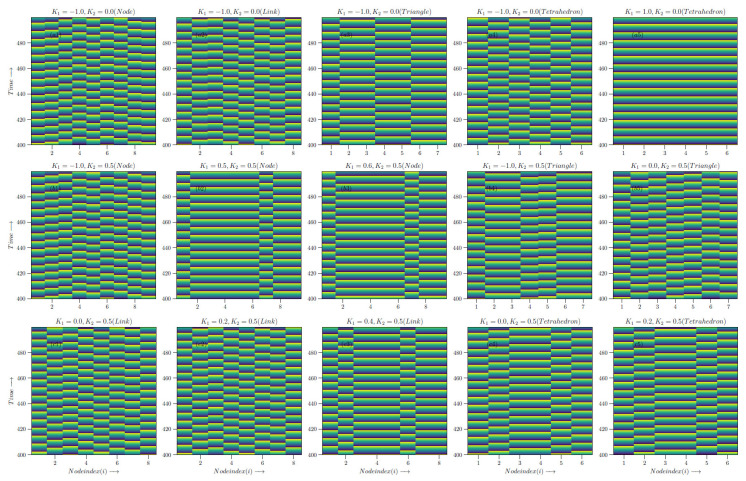
The time evolution of each node $\theta_{i}\left(t\right)$ vs. *t* at different $K_{1}$ and $K_{2}$ values, as marked by diamonds, stars and crosses in Figure 6.

The patterns shown in the two-simplex case can be deciphered more easily via a space–time plot. These were plotted between iterates 400 and 500 after discarding a transient of 400 iterates, where the patterns had stabilized. Following the same sequence as above, the following can be obtained:

1. Crosses ($K_{2}=0.0,K_{1}=-1.0$): The patterns here can be seen in the first row of Figure 10. The figure shows the behavior at each node for two simplex situations sharing nodes, links, triangles and tetrahedra. The lead and lag patterns can be seen in the space–time plot. The phase angles at nodes labeled 1 to 4 follow node 4 which leads, while node 5 lags and node 6 is synchronized with node 4, which is ahead of nodes 7 to 10. For the shared link case, nodes 1 and 5 are synchronized and the remaining nodes are desynchronized. The triangle shared case shows three synchronized clusters of two nodes and a single node, none of which are in step with each other. The shared tetrahedra can be fully synchronized ($K_{1}=-1.0$) or fully desynchronized ($K_{1}=1.0$).
2. Stars ($K_{2}=0.5;K_{1}=-1,0.4$, and 0.5): The shared node cases can be seen in the second row of the space–time plot. As mentioned above, the $K_{1}=-1.0$ node linked case is multi-clustered, with nodes 4 and 7 being synchronized with each other. The remaining nodes show no systematic behavior. The $K_{1}=0.5$ case shows three clusters: nodes 2,4,56 are synchronized nodes, 1 and 7 are synchronized and nodes 9 and 8 are synchronized. The $K_{1}=0.6$, $K_{2}=0.5$ node linked case is similar. The two shared cases denoted by triangles are seen in the same row. Here, of the $K_{1}=-1$ nodes 2,3,6,7 are in step, 1 and 4 are in step and node 5 evolves independently. For $K_{1}=0.0$, no clear pattern is seen though there are two clusters.
3. Diamonds: ($K_{2}=0.5;K_{1}=0,0.3$, and 0.4 case, link shared). This is the last row in the space–time plot. Here, there are three link shared cases and two tetrahedra shared cases. For $K_{1}$=0.0, $K_{2}=0.5$, (link shared) node 5 leads all the other nodes. For $K_{1}=0.2$ and $K_{2}=0.5$, nodes 2,6 are in step, as are nodes 3,8 and 1,5. For links $K_{1}=0.4$, $K_{2}=0.5$, there are two clearly synchronized clusters of nodes 1,3,4,5,7,8 and nodes 2,6. For the shared tetrahedra, we can observed a synchronized cluster where 1,5 nodes are in step, 3,4 are in step and 2,6 are in step (three clusters). For the last case, again, there are three clusters in step: 3,4, 1,5 and 2,6.

The observed patterns are due to cluster synchronization and lead to partially synchronized values for the ordered parameter. These appear to be stable, at least during the period of analysis. Further eigenvalue analysis needs to be carried out to identify the bifurcation behavior that leads to these.

## 6. Summary and Conclusions

We explored two aspects of the dynamics of simplicial structures: the role of the simplicial structure of the substrate and the role of the higher-order interactions. Some recent studies on higher-order interactions include [48,49]. We note that both features make significant contributions to the qualitative features of the dynamics and the transition to synchronization. The substrate structure was controlled via a growing algorithm which incorporates geometric compatibility and chemical structure, leading to compact, mixed and sparse structures. We note that compact structures have a greater tendency to synchronize and to show abrupt synchronization. This also depends on the distribution of the frequencies of the oscillators. We also note that the paths to synchronization are achieved via cluster synchronization, with the exact paths being dependent on the structure of the lattice. Some structures show partial synchronization due to the presence of specific dynamical states. This can depend on the eigenvalue distribution. Similar behavior can be seen in spin systems.

The hysteresis phenomenon allows for a very stable response to synchronization in the reverse phase. In the context of phase locked systems, this could permit very stable synchronization and frequency-locking on the reverse phase, even if individual oscillators change frequencies.

In the context of small systems, we note the phase shifts between individual oscillators and that the system geometries control how shared nodes lead to synchronization between subsets of oscillators with different node labels. This could be put to use for message encryptation purposes.

Noise effects can be very important in these systems, and have not been widely explored. Individual nodes can be more or less stable to noise, depending on the number of shared neighbours. Intermittency effects are possible in such systems.

Two-cluster and single-cluster configurations can also be utilized to switch between frequencies, contributing to resonance effects. Further investigation could lead to quite useful applications. Our other results may have relevance to studies of nanostructures and the human connectome.
